# Cortical Desmoid of the Distal Femur—Incidentaloma or Insertional Tendinopathy?

**DOI:** 10.3390/jcm12082969

**Published:** 2023-04-19

**Authors:** Bastian Mester, Manuel Burggraf, Paula Beck, Heinz-Lothar Meyer, Christina Polan, Thomas Albrecht, Wiebke Guder, Arne Streitbürger, Marcel Dudda, Jendrik Hardes

**Affiliations:** 1 Department for Trauma, Hand and Reconstructive Surgery, University Hospital Essen, Hufelandstraße 55, 45147 Essen, Germany; 2 Department for Tumor Orthopedics, University Hospital Essen, Hufelandstraße 55, 45147 Essen, Germany; 3 Department for Orthopedics and Trauma Surgery, BG-Klinikum Duisburg, University of Duisburg-Essen, Großenbaumer Allee 250, 47249 Duisburg, Germany

**Keywords:** cortical desmoid, DFCI, competitive sports, bone tumor, insertional tendinopathy

## Abstract

Background: The cortical desmoid (DFCI) of the posteromedial femoral condyle is considered an asymptomatic incidental finding in adolescents without clinical relevance. The aim of this study was to evaluate the clinical relevance of DFCI from both a tumor orthopedic and sports medicine point of view. Methods: *n* = 23 patients (13.74 ± 2.74 years; nineteen female, four male) with DFCI of the posteromedial femoral condyle were included. A localized posteromedial knee pain on exertion was differentiated from non-specific knee pain. Symptom duration, additional pathologies, number of MRIs, sports activity and training intensity, downtime, therapeutic modalities, and relief/remission of symptoms were documented. The Tegner activity scale (TAS) and Lysholm score (LS) were collected. The influence of specific posteromedial pain, MRI presence of paratendinous cysts, sports level as well as physiotherapy on downtime and LS/TAS was statistically analyzed. Results: 100% reported knee symptoms at initial presentation. A localized posteromedial pain was documented in 52%. In 16/23 (70%) additional functional pathologies were diagnosed. Patients were physically highly active with high training intensities (6.52 ± 5.87 h/week) and performance level (65% competitive vs. 35% recreational). Patients underwent 1.91 ± 0.97 MRIs (max four). Symptom duration was 10.48 ± 11.02 weeks. A follow-up examination was performed after 12.62 ± 10.41 months (*n* = two lost to follow-up). 17/21 had physiotherapy, on average 17.06 ± 13.33 units. Overall downtime was 13.39 ± 12.50 weeks, the return-to-sports (RTS) rate 81%. 100%/38% reported a relief/remission of complaints. LS was 93.29 ± 7.95, median TAS before onset of knee complaints/at follow-up 7 (6–7)/7 (5–7). Specific posteromedial pain, presence of paratendinous cysts, sports level and physiotherapy had no significant influence on downtime and outcome parameters (n.s.). Conclusions: DFCI as a pathognomonic finding is recurrently encountered in the MRIs of children and adolescents. This knowledge is essential to spare patients from overtreatment. Contrary to the literature, the present results implicate a clinical relevance of DFCI particularly in those who are physically highly active with localized pain on exertion. Structured physiotherapy as basic treatment is recommended.

## 1. Introduction

The cortical desmoid, in the literature also referred to as distal femoral cortical irregularity (DFCI) or cortical irregularity syndrome, is a common finding in radiological diagnostics of the knee joint [1,2]. DFCI is considered a benign, self-limiting fibrous or fibro-osseous lesion that most frequently occurs in the posteromedial aspect of the femoral condyles or more unusually at the aponeurosis of the adductor magnus muscle [2,3]. On plain radiographs, DFCI appears as a small radiolucent area with surrounding sclerosis.

As different and partially misleading terms are used in nomenclature, it is crucial to differentiate DFCI from an entity referred to as “desmoid tumor” or “aggressive fibromatosis”, defined as an aggressive (myo)fibroblastic neoplasm with an infiltrative growth and a tendency to local recurrence [4,5].

Traditionally, the overall incidence of DFCI is reported defensively between 3.6% and 11.5% and an age peak of 10–15 years [6,7]. As an increasing number of magnetic resonance imaging (MRI) for different indications is conducted even in adolescents and children of younger age over the last decades, DFCI is incidentally observed more often due to the higher sensitivity of MRI compared to plain radiographs [8].

Recently, MRI cross-sectional studies on a relevant number of asymptomatic competitive alpine skiers revealed dramatically higher incidences between 58% and 63% [2,9,10], implicating an accumulation of DFCI in ambitious and high-level athletes.

Subsequently, an insertional tendinopathy of the medial head of the gastrocnemius or adductor magnus muscle due to repetitive mechanical stress at the tendinous attachment sites (“tug lesion”) is discussed as a possible pathogenetic explanation [2,3,11]. MRI morphology also reveals signal alterations of the determined tendon attachments itself as well as cyst-like formations adjacent to the DFCI [2]. An association to acute trauma has also been discussed [11]. Despite this, DFCI is widely regarded as an asymptomatic incidental MRI finding without any clinical relevance. It must be considered that the available studies investigating a relevant number of patients focus on epidemiologic and MRI-morphologic aspects in asymptomatic patient cohorts. Very few case reports correlate symptomatic patients with recurrent knee pain to the MRI finding of DFCI [3,9].

The aim of this study was to evaluate a clinical relevance of DFCI on a cohort of young patients with load-dependent knee pain and proof of DFCI of the posteromedial femoral condyle on MRI, from both a tumor orthopedic and sports medicine point of view.

## 2. Materials and Methods

For this retrospective single-center study, the local patient database was screened by software-assistance for the occurrence of the tags “desmoid”, “cortical desmoid”, “cortical irregularity”, “distal femoral cortical irregularity”, cortical irregularity syndrome” and “DFCI” between January 2016 and December 2022.

The search revealed 109 hits, leaving 59 after filtering for duplicates. All correspondences were screened for relevance in terms of content, identifying *n* = 23 patients for study inclusion. Patients were presented with recent MRI in the outpatient department for tumor orthopedics due to insecurity about the entity of the MRI findings and to exclude a malignant bone tumor. In all cases, the diagnosis of DFCI was confirmed by recent MRI and its pathognomonic morphology.

**Figure 1:**
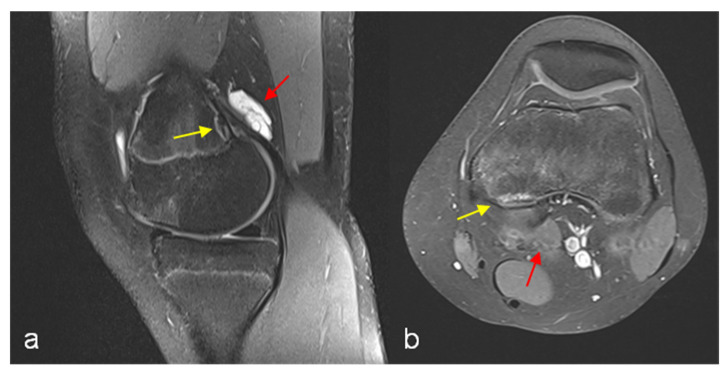
MRI left knee PD TSE FS sagittal reconstruction (**a**) and T1 TSE FS (Gd contrast agent) transversal reconstruction (**b**). Cystic fluid-filled lesion above the femoral origin of the medial gastrocnemius head with marginal Gd enhancement (red arrows), discrete signal enhancement at the tendinous insertion site. Cortical irregularity (DFCI) with signal enhancement and low-signal marginal sclerosis (yellow arrows).

The finding of DFCI was defined as a circumscribed, usually oval area of high signal intensity expanding into bone on fat-suppressed proton density or T2-weighted MRI scans, usually with a thin dark rim at the periphery representing sclerosis [2]. The presence of an accompanying paratendinous cystic lesion is optional (Figure 1).

Digital MRIs were re-scanned by both a radiologist experienced in musculoskeletal imaging and a sports trauma and/or tumor surgeon and diagnosis was confirmed.

Ethical approval was obtained from the local ethical committee (No. 22-10550-BO). The study was performed in accordance with the guidelines of the World Medical Association Declaration of Helsinki. The requirement for acquisition of informed consent from the patients was waived because of the retrospective nature of the study.

All demographic, clinical and radiological data were retrieved from the correspondences available in the local patient database. Missing values were complemented by inquiry in the context of routine clinical control examinations and telephone interviews.

Demographic characteristics were recorded from the database. Regular type and level of preferred sports activity (competitive vs. recreational) as well as frequency (training hours per week) were documented.

On initial presentation, a localized specific posteromedial exertional pain was differentiated from non-specific knee pain of other or multiple localizations using analysis of complaints and subtle clinical examination. The intervals between beginning of complaints and diagnosis as well as the interval between first MRI with proof of DFCI and first clinic presentation were calculated. Accompanying both structural and functional pathologies were inquired.

Regarding radiological parameters, the total number of MRIs and application of X-ray (conventional, CT-scans) were documented. MRI results were screened for accompanying paratendinous cyst-like formations (yes/no) for every single patient.

A follow-up examination was performed to evaluate the course of treatment and effect of interventions. Total downtime for the preferred sports activity due to knee complaints was documented. The number and duration of physiotherapy units as well as adjuvant alternative treatment modalities were counted. For outcome measurement, patients were asked for relief (yes/no) or remission of complaints (yes/no) and time elapsed until return-to-sports (RTS). Furthermore, the Lysholm score (LS) and Tegner activity scale (TAS) were recorded [12,13] to measure knee function and level of activity in this physically active patient cohort. The LS was initially designed for physician administration and was validated in patients with ACL injuries and meniscal injuries, but also has been validated as a patient-administered instrument to measure symptoms and function in patients with a variety of knee pathologies. TAS is a scale that aims to provide a standardized method of grading work and sporting activities and was developed to complement the LS [14,15].

Furthermore, the influence of the presence/absence of specific posteromedial knee pain and of paratendinous cysts in MRI as well as level of sports activity (competitive vs. recreational) on downtime and outcome parameters (LS, TAS) was evaluated. The influence of structured physiotherapy on downtime and LS/TAS was additionally analyzed.

**Figure 2:**
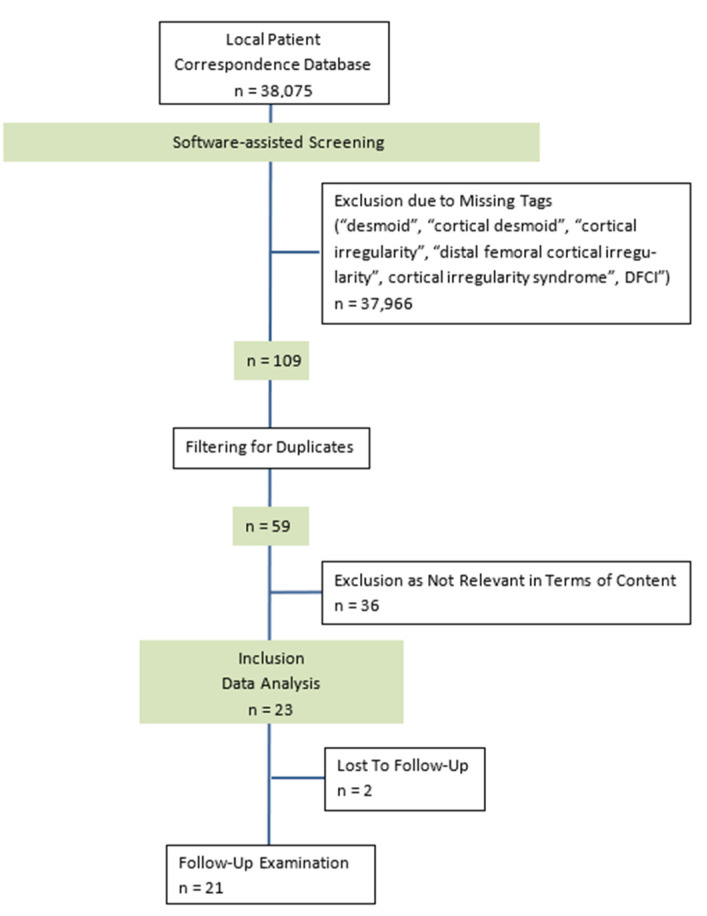
CONSORT diagram presenting the enrolment of the final study population according to [16]. Inclusion and data analysis of *n* = 23 patients; follow-up examination available for *n* = 21 patients (*n* = 2 lost to follow-up).

The patient’s enrolment process according to CONSORT criteria is shown in Figure 2 [16].

### Statistical Analysis

A detailed descriptive epidemiological analysis was conducted including arithmetic mean, standard deviation, minimum, maximum, median at continuous data and scores as well as relative frequency for explained variables.

Statistical analysis was performed using Excel^®^ for Microsoft 365 MSO (version 2205; Microsoft Corp., Redmond, WA, USA) and GraphPad Prism Software (version 9.5.0 for MacOS, San Diego, CA, USA). Due to the number of cases, no normal distribution was assumed and the Mann–Whitney-U test was used for subgroup comparison. Results are expressed as median. The correlation of individual variables was calculated using the Spearman rank correlation coefficient r with 95% confidence interval.

Significant differences were assumed for a *p* < 0.05 (two-tailed). Data analysis was strictly exploratory and no adjustment was made for multiple testing. Graphical representation is as a scatter dot plot with each point reflecting a data value and the horizontal line reflecting the median.

## 3. Results

Basic demographic data, information regarding sports activity and downtime as well as data regarding clinical presentation and radiological diagnostics were available for all *n* = 23 (100%) patients. As two patients did not attend clinical control examinations (=lost to follow-up), data regarding therapies, course of complaints, RTS and outcome parameters could be evaluated for *n* = 21 (91%) patients.

The mean age of the cohort was 13.74 ± 2.74 (min 8, max 21) years. The included patients were predominantly female with a distribution female to male = 19:4.

100% had knee symptoms in terms of knee pain (*n* = 14 left, *n* = 9 right knees) at the time of first presentation. In 52% of the cases (*n* = 12), an additional or isolated localized posteromedial pain on exertion of the knee could be documented by clinical examination.

In a relevant number of cases (*n* = 16, 70%), at least one additional functional pathology was diagnosed: *n* = five pelvic torsion and hamstring tightness, *n* = five knee distorsion, *n* = four iliotibial band syndrome and pes anserine tendinopathy, *n* = two patellofemoral pain syndrome, *n* = one neuromuscular disbalancing and *n* = one habitual knee hyperlaxity.

Structural accompanying pathologies were excluded regularly by MRI in 74% (17/23) of cases. Within the remaining 26% (6/23), *n* = three had an unspecific bone marrow edema of the femoral condyles or tibial plateau, *n* = two showed an increased signal intensity of the medial meniscus without MRI proof of meniscal tear, and *n* = one joint effusion. In 39% of all cases (9/23), MRI revealed a DFCI-accompanying paratendinous cyst at the insertion site of the medial head of the gastrocnemius muscle.

All patients were doing sports on a regular basis before the onset of knee complaints. The most common preferred sports activities were swimming (30%), dancing/ballet (26%) and field hockey (13%); multiple answers were possible. Above average, patients were highly physically active with a mean training intensity of 6.52 ± 5.87 (min 1, max 24) hours/week, having pursued their favorite sports for 7.23 ± 3.79 (min 2, max 15) years. Sixty-five percent stated to practice the sports activity on a competitive or even professional level and 35% as recreational sports.

Data including clinical presentation and accompanying pathologies as well as prevalence of paratendinous cystic lesions are presented in Table 1; information regarding sports activity and level before onset of complaints are presented in Table 2.

65% of patients (*n* = 15) underwent an initial conventional X-ray in two planes of the affected knee as a primary diagnostic tool. All patients received MRI diagnostics for their final diagnosis as well as monitoring of the DFCI. On average, up to four MRI investigations (mean 1.91 ± 0.97; min one, max four) were performed at the beginning and in the course of the treatment. An additional CT scan was performed for diagnostic reasons in 3/23 (13%) patients.

Mean symptom duration to initial presentation in our clinic was 10.48 ± 11.02 (min 3, max 52) weeks. The mean interval between the date of initial MRI and first contact in our outpatient department with confirmation of DFCI was 64.74 ± 85.25 (min 6, max 304) days.

The clinical follow-up examination was performed 12.62 ± 10.41 months (min 3, max 44) after initial presentation; an evaluation could be completed for *n* = 21 patients.

Structured physiotherapy as basic therapeutic modality was recommended after initial presentation in our clinic to all patients. 17/21 (81%) stated to have finally undergone physiotherapeutic exercises for their knee complaints until follow up. On average, patients had 17.06 ± 13.33 (min one, max sixty) physiotherapy units, over a mean period of 17.41 ± 16.86 (min 1, max 72) weeks. The following adjuvant alternative treatment modalities had been selected in 18/21 (86%) patients: *n* = eleven bandage / taping, *n* = five focused ultrasound / extracorporeal shockwave, *n* = three insoles, *n* = two electrotherapy and *n* = one intraarticular injection of hyaluronic acid. *n* = two patients underwent open surgical resection of a paratendinous cystic lesion and debridement of the tendinous insertion at the site of DFCI due to persistent knee complaints.

The overall downtime due to knee complaints within the patient cohort was 13.39 ± 12.50 (min 2, max 48) weeks. *n* = four patients did not return to their previously preferred sports activity, resulting in an overall RTS rate of 81%. All patients were able to report a relief in complaints, whereas 38% (8/21) reported a complete remission.

Regarding the subjective functional outcome, the mean LS was 93.29 ± 7.95 (min 72, max 100). Median TAS before onset of knee complaints was estimated 7.00 (6.00–7.00) vs. 7.00 (5.00–7.00) at the time of follow-up examination.

**Figure 3:**
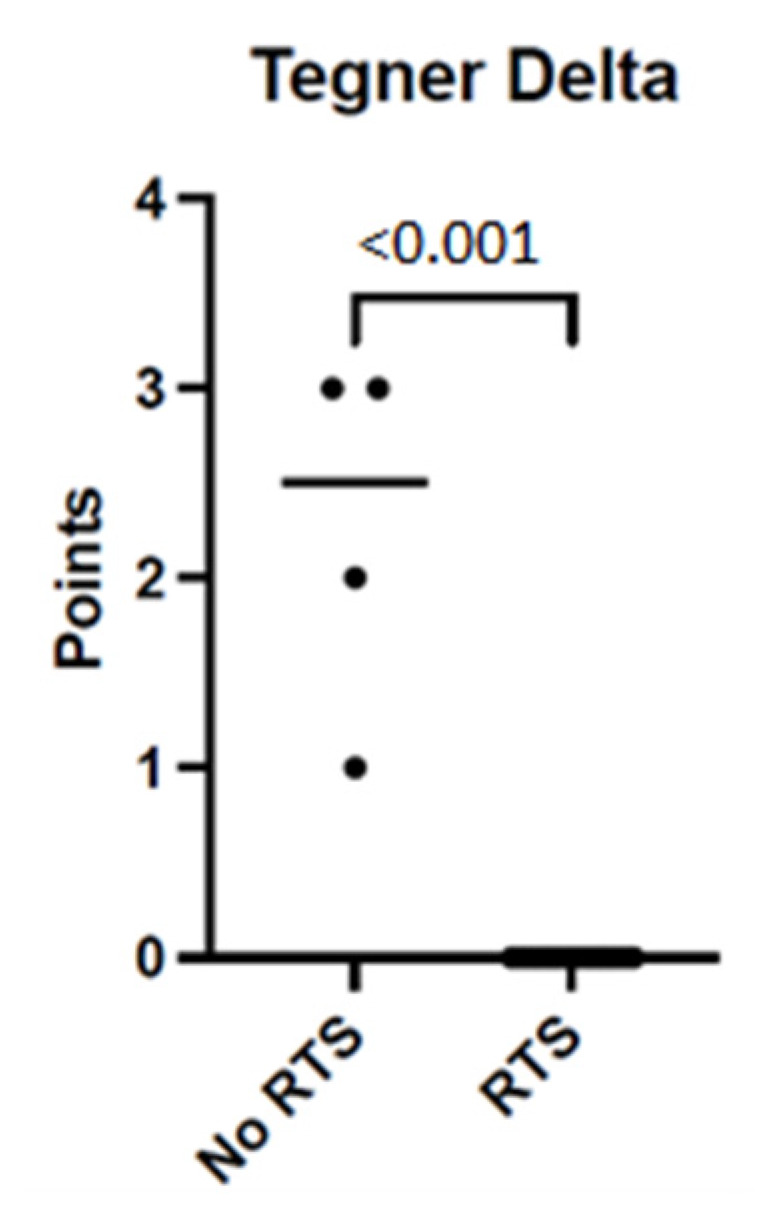
Median loss of activity according to Tegner activity scale, no RTS vs. RTS. Scatter plot. Significant difference in median Tegner Delta (points) for patients with no return to the preferred sports activity vs. patients who accomplished RTS until follow up, *p* < 0.001. RTS = return-to-sports.

Patients who stated not having returned to their preferred sports activity until their follow-up examination (*n* = 4, 19%) had a highly significant loss of activity regarding TAS compared to patients who accomplished an RTS (TAS-delta 2.5 [1.25–3] for no RTS vs. 0 [0–0] for RTS; *p* < 0.001, Figure 3).

The descriptive results regarding downtime, RTS and functional outcome parameters are given in Table 3.

**Figure 4:**
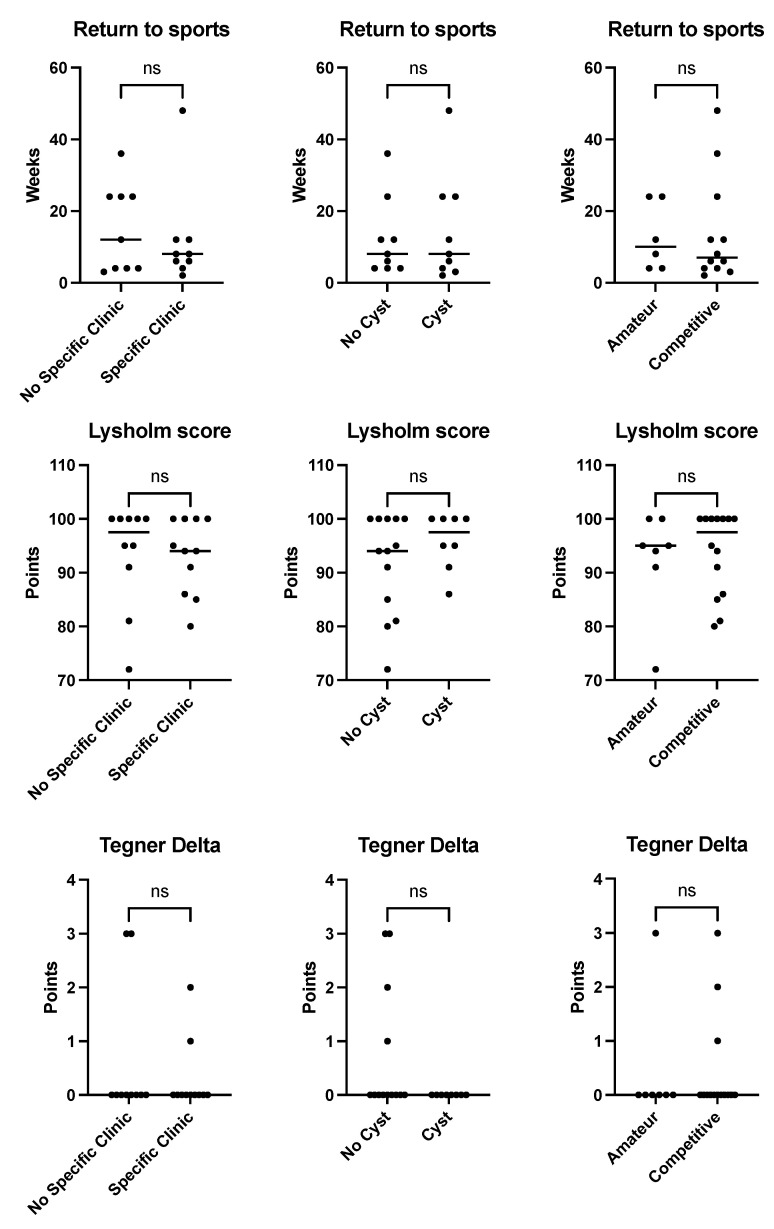
No influence of clinical presentation, MRI morphology of DFCI nor sports level on downtime and outcome parameters. Scatter plots. Influence of absence/presence of a specific posteromedial knee pain, paratendinous cystic lesion (MRI) or level of sports activity on time to RTS (=downtime), LS and loss of activity (TAS delta) not demonstrated in this study, *p* > 0.05. LS = Lysholm score; TAS = Tegner activity scale; MRI = magnetic resonance imaging. ns = not significant.

An influence of the presence/absence of a specific posteromedial knee pain and of paratendinous cysts in MRI as well as the level of sports activity on total downtime and outcome parameters (LS, TAS) could not be proved (n.s. for all values, Figure 4).

Duration of physiotherapy and number of units had no statistical impact, neither on total downtime nor on LS and TAS (n.s. for all values).

## 4. Discussion

The main goal of this study was to demonstrate the clinical relevance of DFCI from a tumor orthopedic as well as sports medicine point of view, investigating a cohort of physically active children and adolescents with activity-limiting knee symptoms and DFCI. As the most important findings, all patient included in this investigation complained about relevant knee pain in a large proportion with specific load pain that could be correlated clinically to the MRI finding of DFCI of the posteromedial femoral condyle. A predominant proportion of patients were highly physically active, supporting the pathophysiological hypothesis of an insertional tendinopathy of the medial head of the gastrocnemius muscle. Non-invasive treatment with specific physiotherapy programs led to relief in symptoms and high return-to-sports rates at a similar level as well as excellent subjective functional outcome. The influence of posteromedial knee pain, paratendinous cysts, level of sports activity and physiotherapy could not be shown.

Already in 1988, Ritschl et al. established the “tug lesion” theory that DFCI may originate as a result of repetitive stress at tendon attachments due to extensive mechanical load [17]. According to this, DFCI is the result of repetitive traction from the medial head of the gastrocnemius muscle at its attachment site on the posteromedial femoral condyle. Apart from the cortical irregularity itself, an increased signal intensity of the tendon as well as paratendinous cystic lesions are observed [2]. This theory is supported by an observation of higher incidence rates of DFCI in physically highly active athletes with frequent activation of the calf muscles, and even in the setting of knee trauma in terms of an acute traction injuries of the medial head of gastrocnemius muscle [11]. In this context, Stern et al. published a single-center secondary MRI analysis of a prospective trial on *n* = 105 asymptomatic competitive adolescent alpine skiers regarding the proof of DFCI on knee MRI, compared to a control group. The incidence of DFCI was 58% in alpine skiers and 27% in control participants; this difference was statistically significant [2]. Another investigation of the same study group on ski athletes showed an even higher incidence of DFCI of 63% [10]. These results are supported by the findings of the present study, as the rate of physically active patients was 100%, with 65% of patients doing sports on a competitive or even professional level. Concordantly, the training intensity was very high in the examined patient cohort. The most common preferred sports activities stated were swimming, dancing/ballet, and field hockey (30%, 26% and 13%, respectively). These sports are characterized by repetitive activation of the calf muscles, also supporting the “tug theory” by Ritschl et al. [17]. The preferred sports activities of patients with DFCI may vary depending on regional opportunities and social background.

In contrast to most patients investigated in the available literature, all patients included in the recent investigation complained about knee symptoms and 52% of the cases had a specific load pain located at the posteromedial corner of the knee. Both alpine skiers as well as control participants in the study by Stern et al. mentioned above were asymptomatic and showed high incidences of DFCI at the same time [2].

Different other authors also evaluate the DFCI of the posteromedial femoral condyle as an incidental finding without clinical relevance [7,18,19]. It must be considered that most studies conducted in the past focused on epidemiological and MRI-morphologic aspects more than on clinical presentation or presence/absence of specific knee symptoms. Fröhlich et al. performed a cross-sectional MRI study on *n* = 108 competitive adolescent alpine skiers on the one hand and interviewing them for overuse-related knee complaints on the other hand [10]. 47% of the patients included suffered from at least one overuse-related knee complaint within twelve months before the MRI examination. Although the most common MRI finding in the study was the DFCI of the posteromedial condyle and 73% of these have been symptomatic according to the interview, the authors could not differentiate a specific posteromedial knee pain anatomically linked to the DFCI. It is concluded that the sensitivity of an MRI-based detection of overuse-related knee complaints regarding an association with corresponding MRI abnormalities is limited.

As the correlation of DFCI with specific clinical symptoms is only based on clinical features and strongly depends on a subtle clinical examination, a misinterpretation of other functional pathologies finally cannot fully be excluded.

Although DFCI is mainly considered an asymptomatic incidental finding without clinical implications in the available literature, there are few case reports presenting patients with specific localized posteromedial knee symptoms linked to the proof of DFCI in MRI. To our knowledge, this is the first systematic investigation establishing a link between knee symptoms and DFCI in a relevant number of physically active children and adolescents.

Kontogeorgakos et al. differentiate the case of a physically active adolescent with localized pain at the inner aspect of the knee proximal to the medial femoral epicondyle and MRI proof of DFCI from three other cases with proof of DFCI, but leading accompanying structural and/or functional knee pathologies [3]. According to the authors, the entity of symptomatic DFCI exists and has a certain significance but must be delineated from knee symptoms for other reasons.

In the present study, functional accompanying pathologies were found in a large proportion (70%). On the other hand, 52% reported a localized posteromedial load pain of the knee. In our experience, it is possible to correlate specific knee symptoms using subtle clinical examination with the MRI finding of DFCI. Especially in combination with a history of high sports activity levels, a localized posteromedial load pain can be considered a reasonably reliable clinical sign for a symptomatic DFCI as its own entity in children and adolescents. Other authors confirm this within single case reports [9,20,21], which is reinforced by the results of this investigation on a relevant patient cohort with symptomatic DFCI.

However, the presence of a specific posteromedial knee pain, MRI-morphologic parameters (paratendinous cystic lesions, 39% in the present study) and pre-existent sports level had no impact on total downtime and outcome parameters in this study. Consequently, these parameters do not seem to have a predictive value regarding the course of disease and functional outcome.

Regarding the outcome following primarily conservatively treated DFCI of the posteromedial condyle in physically active children and adolescents, we could demonstrate relief of complaints in 100%, but a complete remission in only 38%. All studies available dealing with this issue agree that DFCI is a benign, self-limiting condition [2,3,7,9,11]. According to this, it can be hypothesized that a larger proportion of patients of this cohort will accomplish a complete remission in the mid- to long-term follow up. On the other hand, these results emphasize that DFCI must be considered a relevant entity causing ongoing knee symptoms over twelve months and downtimes in sports activities for several weeks. The results of LS and TAS show that excellent functional outcomes and an RTS at equivalent levels can be expected. Though, a measurable influence of the implementation of structured physiotherapy on downtime and LS/TAS was not demonstrated in this investigation. It may be hypothesized that an effect could be shown in the setting of a larger patient cohort and more statistical power, as it has a proven positive effect in conservative treatment programs of other insertional tendinopathies [22].

To our knowledge, there is no comparable study measuring the functional outcome after primarily conservative treatment for DFCI. Future prospective studies comparing the results of conservative treatment vs. “watchful waiting” are desirable. As 81% of patients in this study received structured physiotherapy (in combination with various adjuvant therapies), according to our results we recommend conservative treatment with physiotherapy as the central therapeutic modality inspired by treatment programs for insertional tendinopathies at other localizations [22]. Surgical resection of the cyst with tendon debridement may be considered cautiously in single selected cases with therapy-resistant complaints and MRI-morphologic alterations (paratendinous cysts, abnormal tendon signal intensity).

Although DFCI shows high incidence rates in young, physically active patients, the entity seems to be still unknown to many sports physicians and even experienced knee surgeons. Due to ignorance or concern, unnecessary further investigations may be conducted [9,21]. In the present study, 65% had an initial X-ray and up to four MRI investigations were performed. Furthermore, *n* = three patients underwent additional CT scans. As a so-called “do-not-touch lesion” with no need for histopathological sampling, DFCI and its MRI morphology must be interpreted correctly and not be misinterpreted as an aggressive osseous neoplasia [2,23]. The differential diagnosis list includes fibrous cortical defects (FCD), non-ossifying fibroma (NOF), osteosarcoma and Ewing’s sarcoma [9,21]. Unfortunately, the nomenclature of DFCI is inconclusive, as it has to be distinguished from the entity of desmoid tumors (=aggressive fibromatosis, [4,5,24]).

It must be considered that most patients included in the present investigation have initially been admitted to the department of tumor orthopedics due to insecurity regarding benignity and treatment for the MRI finding of DFCI. Consequently, the need for wider education of pediatricians, sports physicians as well as knee surgeons is evident.

The authors’ diagnostic and therapeutic algorithm include interpretation of initial plain radiographs, if applicable, and knee MRI as primary radiological diagnostic means, sports medical history of the patient and correlation to subtle clinical examination. Conservative treatment (structured physiotherapy) is initiated, patients are followed up for clinical re-evaluations within three to six months. In less apparent cases (differential diagnosis) or persistent complaints, we recommend MRI follow up six months after initial MRI; routine follow-up examinations are not required.

**Figure 5:**
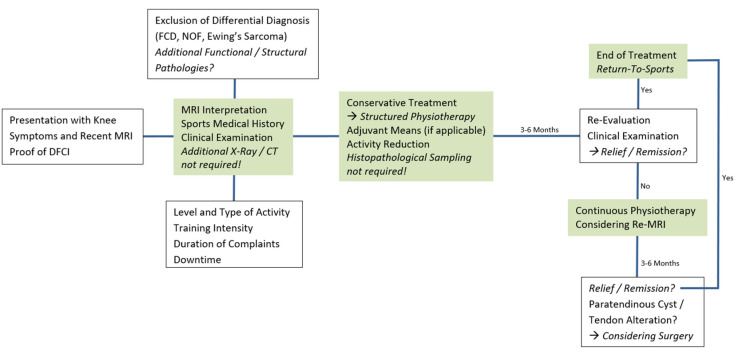
Diagnostic and therapeutic algorithm for DFCI in physically active children and adolescents. The authors’ approach to patients with knee symptoms and MRI proof of DFCI. MRI = magnetic resonance imaging. DFCI = distal femoral cortical irregularity. FCD = fibrous cortical defect. NOF = non-ossifying fibroma. CT = computed tomography.

The authors’ approach to symptomatic DFCI in children and adolescents is shown in Figure 5.

### Limitations

Limitations of this investigation are the retrospective design and the limited number of patients. This is attributable to the distinct selection of patients with symptomatic DFCI, which is—to our knowledge—reported systematically for the first time in a coherent cohort of patients. A sample size calculation was not performed. Results of the explorative statistical analysis must be interpreted carefully due to the low number of patients and consequent lack of statistical power. Furthermore, a control group regarding the evaluation of clinical outcome and RTS after DFCI is missing.

## 5. Conclusions

Knowledge of DFCI as a pathognomonic image finding is essential to spare the patient from overinvestigation and overtreatment. Contrary to the data available to date, it appears that—particularly in the physically highly active patient with localized pain on exertion—DFCI seems to be clinically relevant. Further clinical studies with larger patient cohorts with DFCI are desirable. Concordant to treatment concepts for other insertional tendinopathies, a structured conservative treatment with physiotherapy as basic therapy for symptomatic DFCI is recommended.

## Figures and Tables

**Table 1:** Presentation and accompanying functional and/or structural pathologies as well as prevalence of paratendinous cystic lesions according to clinical examination and MRI for every single patient (*n* = 23).

Patient	Posteromedial Pain	Functional Pathology	Structural Pathology	Paratendinous Cyst
1	No	Distorsion		No
2	Yes	PFPS, PT/HS		No
3	Yes	PT/HS		No
4	Yes	NM Disbalance		Yes
5	No	Distorsion	Femoral BME	Yes
6	No	ITBS		No
7	No	ITBS		No
8	No	Hyperlaxity		No
9	Yes			No
10	Yes	Distorsion		No
11	Yes	PT/HS		Yes
12	No		Tibial BME	No
13	Yes			No
14	Yes		Joint Effusion	Yes
15	No	PFPS	Femoral BME	No
16	No			Yes
17	Yes	ITBS		Yes
18	No	Distorsion		No
19	Yes	PAT	Hyperintense MM	Yes
20	No	PT/HS		No
21	Yes			No
22	Yes		Hyperintense MM	Yes
23	No	Distorsion, PT/HS		Yes

PT/HS = pelvic torsion hamstring tightness, ITBS = iliotibial band syndrome, PAT = pes anserine tendinopathy, PFPS = patellofemoral pain syndrome, NM = neuromuscular, BME = mone marrow edema, MM = medial meniscus.

**Table 2:** Preferred sports activity, intensity and performance level before onset of complaints for every single patient (*n* = 23).

Patient	Preferred Sports	Practice (y)	Training (h/Week)	Level
1	Field Hockey	11	9	Professional
2	Dancing, Martial Art	mv	mv	Recreational
3	Swimming, Ballet	10	10	Competitive
4	Swimming	14	20	Professional
5	Swimming	10	4.5	Competitive
6	Soccer	3	3	Recreational
7	Handball	8	12	Competitive
8	Gymnastics	3	1	Recreational
9	Dancing	7	2	Competitive
10	Soccer	6	2	Recreational
11	Swimming, Soccer	2	2	Recreational
12	Field Hockey	10	8	Professional
13	Handball	9	3	Competitive
14	Gymnastics	15	5	Competitive
15	Swimming, Ballet	2	4	Recreational
16	Riding, Martial Art	2	2	Recreational
17	Field Hockey	10	3	Competitive
18	Dancing	4	3	Competitive
19	Swimming	7	24	Competitive
20	Figure Skating	8	3	Competitive
21	Dancing, Volleyball	9	5	Recreational
22	Swimming	7	12	Competitive
23	Volleyball, Tennis	2	6	Competitive
Mean ± SD		7.23 ± 3.79	6.52 ± 5.87	

y = years, h = hours; mv = missing value, SD = standard deviation.

**Table 3:** Downtime in preferred sports, RTS as well as results of LS and TAS at follow up for every single patient (*n* = 23). Incomplete data for patients no. 2 and no. 5 are lost to follow up.

Patient	Downtime (Weeks)	RTS	LS	TAS Pre	TAS Post
1	4	Yes	91	9	9
2	mv	mv	mv	mv	mv
3	No RTS	No	85	7	5
4	48	Yes	86	7	7
5	24	mv	mv	mv	mv
6	No RTS	No	72	7	4
7	36	Yes	100	7	7
8	4	Yes	100	5	5
9	No RTS	No	94	6	5
10	8	Yes	100	4	4
11	4	Yes	91	7	7
12	12	Yes	100	9	9
13	6	Yes	80	7	7
14	12	Yes	100	7	7
15	24	Yes	95	6	6
16	24	Yes	95	7	7
17	2	Yes	100	9	9
18	4	Yes	81	6	6
19	8	Yes	95	7	7
20	No RTS	No	100	7	4
21	12	Yes	94	6	6
22	6	Yes	100	7	7
23	3	Yes	100	7	7
Mean ± SD	13.39 ± 12.50		93.29 ± 7.95	6.86 ± 1.17	6.43 ± 1.50

RTS = return to sports; LS = Lysholm score; TAS = Tegner activity scale; mv = missing value; SD = standard deviation.

## Data Availability

The data presented in this study are available on reasonable request from the corresponding author.
